# Predicting Response to Brain Stimulation in Depression: a Roadmap for Biomarker Discovery

**DOI:** 10.1007/s40473-021-00226-9

**Published:** 2021-02-15

**Authors:** Camilla L. Nord

**Affiliations:** MRC Cognition and Brain Sciences Unit, University of Cambridge, 15 Chaucer Road, Cambridge, CB2 7EF UK

**Keywords:** Brain stimulation, Depression, Biomarkers, Predicting response, TMS, tDCS

## Abstract

**Purpose of Review:**

Clinical response to brain stimulation treatments for depression is highly variable. A major challenge for the field is predicting an individual patient’s likelihood of response. This review synthesises recent developments in neural predictors of response to targeted brain stimulation in depression. It then proposes a framework to evaluate the clinical potential of putative ‘biomarkers’.

**Recent Findings:**

Largely, developments in identifying putative predictors emerge from two approaches: data-driven, including machine learning algorithms applied to resting state or structural neuroimaging data, and theory-driven, including task-based neuroimaging. Theory-driven approaches can also yield mechanistic insight into the cognitive processes altered by the intervention.

**Summary:**

A pragmatic framework for discovery and testing of biomarkers of brain stimulation response in depression is proposed, involving (1) identification of a cognitive-neural phenotype; (2) confirming its validity as putative biomarker, including out-of-sample replicability and within-subject reliability; (3) establishing the association between this phenotype and treatment response and/or its modifiability with particular brain stimulation interventions via an early-phase randomised controlled trial RCT; and (4) multi-site RCTs of one or more treatment types measuring the generalisability of the biomarker and confirming the superiority of biomarker-selected patients over randomly allocated groups.

## Introduction

The past forty years have revolutionised our understanding of the neural circuitry of depression. Concurrently, developments in neuromodulation have produced techniques to target specific brain circuits non-invasively or with minimal invasiveness. A new field has emerged from these two developments, aiming to treat depression using targeted brain stimulation. A plethora of neuromodulation techniques have now been tested as putative depression interventions, with variable success. The most common non-invasive approaches are various forms of repetitive transcranial magnetic stimulation (rTMS, including theta burst stimulation (TBS)), transcranial direct current stimulation (tDCS), electroconvulsive therapy (ECT), and magnetic seizure therapy (MST) (see [1••] for a recent overview of each type and their comparative clinical efficacy); the most common invasive approach is deep brain stimulation (DBS) (see [2] for a recent review).

In some cases, brain stimulation is a highly effective intervention for depression, even in patients resistant to other treatment approaches [3–6]. But in others, little or no improvement is seen. This is apparent in the large variability in outcomes (or in some cases null results) reported in randomised controlled trials (RCTs) of various brain stimulation interventions [7, 8••, 9••], as well as notable variability in response to brain stimulation for other purposes, including across motor [10, 11] and cognitive [12] systems. This variability is not unique to brain stimulation interventions: It is the norm across all depression treatments. As yet, there are no established techniques to predict treatment response in depression, whether following brain stimulation, antidepressant medication, psychological therapy, or any other intervention. This paper aims to review neural and biological predictors of response to brain stimulation in depression, before proposing a framework for future studies to identify and test potential neural predictors of brain stimulation response.

There is a large and growing field of biomarker development to predict response to depression treatments. The term Predictive ‘biomarker’ is used here to refer to a broad array of measures obtained at baseline (including blood, brain imaging, or cognitive markers) that might predict response to a particular intervention. A subset of these measure neural function using neuroimaging to predict treatment response for pharmacological and psychological treatments (e.g. [13–17, 18•]). Neural predictors might be particularly useful for brain stimulation interventions, which directly perturb the activity within neural circuits, versus indirect perturbation caused by psychological or pharmacological interventions. Ideally, establishing neural predictors of brain stimulation response might simultaneously provide a clearer window on the neural mechanisms of brain stimulation interventions.

The majority of recent efforts to predict the outcome of brain stimulation focus on two neural measures in particular: neuroanatomical location and baseline activity state of the site targeted. In this review, key recent efforts to use anatomical and functional neural measures as putative predictive biomarkers of treatment response will be outlined, focussing primarily on stimulation approaches that attempt to target particular neural regions (i.e. rTMS/TBS, DBS, and tDCS; see [1••] for a recent meta-analysis of all non-surgical brain stimulation in depression, including ECT and MST). Based on recent developments, a pragmatic framework for discovery of specific predictors of brain stimulation response in depression will be proposed, focussing on how studies should test the validity, reliability, and specificity of novel putative biomarkers.

## Taking Neuroanatomical Variation Into Account

A key contributor to variability in response to brain stimulation is individual differences in neuroanatomy. For instance, the most common target for non-invasive brain stimulation interventions is the left dorsolateral prefrontal cortex (DLPFC). To localise the DLPFC, most TMS trials localise the finger region of the primary motor cortex and move five to six centimetres anterior. This approach has been successful in a number of trials [3, 19, 20]. Nevertheless, it leads to vast between-subject differences in the precise neuroanatomical region targeted [21]. The DLPFC is not a homogenous region [22, 23], so even minor differences in site localisation could substantially change the behavioural and clinical effects of perturbation. How successfully brain stimulation targets a given neural site may be one contributor to a patient’s likelihood to respond to brain stimulation.

One solution (already employed to some degree by many studies) is imaging-based localisation. In brain stimulation studies in other cognitive domains, ever-more precise neuroanatomical targeting leads to increasingly larger behavioural effects of brain stimulation. In an elegant demonstration of this effect in healthy controls using parietal TMS, Sack and colleagues tested four different approaches to localisation: the 10–20 electrode scalp system, magnetic resonance imaging (MRI)-based neuronavigation, functional MRI (fMRI)-based localisation using standardised coordinates from the literature, and fMRI neuronavigation based on an individual’s fMRI data [24]. They demonstrated that localisation approach dramatically altered the effect size of behavioural changes evoked by TMS. Subsequent power analyses showed that while 47 participants would have been required to detect the size of the behavioural effect obtained from 10 to 20 localised TMS, only 13 would be required for standardised coordinate-based neuronavigation; only 9 required when using individual MRI-guided neuronavigation; and 5 participants were sufficient to reveal a significant behavioural effect when using individual fMRI-guided TMS neuronavigation. This strongly suggests that improved site localisation in TMS for depression could improve the likelihood of a patient responding.

There is preliminary evidence supporting the utility of better localisation in depressed patients: a patient’s likelihood of responding to rTMS is increased when the site stimulated is more lateral and anterior [25]. Precise, subject-specific localisation (as in the Sack study) could further improve a patient’s likelihood of response. However, the targeted site is only one parameter of many in a given brain stimulation montage. There may also be a particular intensity, coil or electrode angle, delivery number, or interval between sessions that maximise a patient’s probability of responding to brain stimulation. The sheer number of modifiable parameters and their possible combinations means that we may not yet know the exact optimal parameters for targeting specific neural regions or circuits, although computational modelling studies of brain stimulation montages go some way toward addressing this [26, 27, 28••].

## Baseline Neural Measures as a Window Into Response Variability

In addition to between-subject differences in neuroanatomical target location, other baseline neural features could alter the likelihood of clinical response—and, potentially, serve as putative neural ‘biomarkers’ of response. A small number of studies have suggested that certain neuroanatomical features might correspond to a patient’s likelihood of response to particular types of brain stimulation. For instance, smaller amygdala volume pre-treatment was associated with better responding to rTMS [29••]. This was speculated to underpin a mechanism of action of rTMS: amygdala volume increased in rTMS responders only [29••] (although note the laterality of this effect differs). Other structural measures like white matter connectivity may also be useful as neural predictors of treatment response. In a small pilot study of probabilistic tractography in patients implanted with DBS electrodes, high connectivity between the DBS contact to the medial PFC was associated with response [30]. However, this result is very preliminary and requires testing in large samples.

The majority of recent proposals for neural biomarkers of brain stimulation response have been functional measures of baseline neural state in a region or regions. For instance, patients administered rTMS to DLPFC subegions with greater resting-state functional anticorrelation with the subgenual anterior cingulate cortex (sgACC) are more likely to respond than those administered TMS to DLPFC subregions with sparse sgACC functional connectivity [31, 32]. This discovery has led to a new proposal for coil placement optimisation: identifying and targeting subject-specific prefrontal sites with the greatest sgACC anticorrelation [31]. This approach (targeting the DLPFC coordinate that is maximally anticorrelated with the sgACC) was recently tested in an open-label rTMS trial which achieved extremely high remission rates in treatment-resistant depressed patients (note that it also had a number of unusual methodological specifications, including number of pulses, intensity, and session spacing) [33]. The efficacy of this approach might derive from modulating the interaction of two spatially and temporally dissociable functional networks at rest, both considered central to the pathophysiology of depression: the central executive network (including the DLPFC), implicated in attentional, working memory, and decision-making processes, and default mode network (tightly coupled with the sgACC), involved in self-referential processes such as rumination [34].

Even after absolutely precise anatomical or functional localisation, different patients with depression may require altogether different interventions, due to the inherent heterogeneity of the disorder. At the symptom level, two patients meeting diagnostic criteria for depression might not share a single criterion in common [35]. At the neural level, even the most robust group-level neural differences between patients with depression and non-depressed controls still vary at the level of the individual. For instance, most targets of non-invasive brain stimulation studies are found in the left DLPFC, where across a number of studies, depressed patients show group-level hypoactivation during working memory tasks compared to non-depressed participants [36, 37•]. Yet within an individual study, not all patients show hypoactivation; depending on the task employed, some studies even report group-level hyperactivation in depressed patients compared to non-depressed controls [38] (potentially due to differences in task difficulty [39]). Even innovative anatomical and functional approaches to optimise TMS stimulation site and stimulation parameters should only be effective for those patients who show aberrant activation at that site (or a closely coupled region) in the first place. Overcoming this problem of heterogeneity involves identification and testing of baseline disease-relevant metrics that could eventually be used for treatment selection—identifying which intervention is most appropriate for an individual patient.

## Optimising Treatment Selection in Brain Stimulation

Outside the brain stimulation field, numerous predictors of response to antidepressant drugs, psychological therapies, or ECT (for example) have been proposed (e.g. [15, 40–42]). Central to all these proposals is the theory that a given treatment may not be suitable for every individual and that some measure at baseline could distinguish those likely to respond from those unlikely to benefit. For instance, non-responders to rTMS of the dorsomedial prefrontal cortex (DMPFC) show markedly higher baseline pessimism, anhedonia, and loss of interest scores on standard clinical assessments [43•], whereas response to DBS is positively associated with anhedonia [44]. This suggests that specific stimulation types (e.g. rTMS versus DBS) may be particularly suited for certain sub-groups of patients with a given diagnosis, presumably related to neural mechanisms targeted by that intervention. Recently, a number of studies have proposed using neural measures obtained at rest to select patients for particular brain stimulation interventions. For instance, baseline sgACC glutamate activity (measured with positron emission tomography) was higher in sgACC DBS responders compared to non-responders [45]. Similarly, for rTMS protocols targeting the DMPFC, higher resting state connectivity between the DMPFC and sgACC in an individual patient was associated with better treatment outcomes [46]. Greater baseline functional connectivity between the orbitofrontal cortex and sgACC was found to distinguish responders from non-responders to DLPFC rTMS [47]. In contrast, an individual patient’s DMPFC connectivity with the thalamus or putamen was inversely associated with clinical improvement [46]. The role of DMPFC-sgACC interplay in in integrating cognitive and affective information may indicate that patients require a degree of preserved executive control over emotional stimuli to support clinical response to rTMS [48]. Furthermore, in a study examining predictors of response to antidepressant medication, the functional connectivity between neighbouring regions the dorsal ACC and sgACC was inversely associated with treatment response [49]. This provides preliminary evidence for a treatment-specific role of medial prefrontal-to-sgACC connectivity, which would be helpful for future treatment selection or personalised medicine approaches.

### Data-Driven Approaches

A particularly useful prospect in personalised medicine is data-driven approaches to enable discovery of discriminating neural features that predict treatment response. In perhaps the most famous demonstration of this approach, Drysdale and colleagues demonstrated high accuracy in predicting DMPFC rTMS response using a machine learning algorithm applied to resting-state fMRI connectivity data, which identified ‘clusters’ with differential responsiveness to the intervention [50••]. However, an important test for this technique is its ability to generate the same clusters in other samples. The original method used (canonical correlation analysis) was later shown to be unable to generalise to a different dataset [51, 52•]; canonical correlation analysis is prone to over-fitting on high-dimensional data like brain scans (identifying associations that exist by chance) [53•] (note that regularisation has now been used to remedy this issue in the original data [54]).

The second challenge of this and similar approaches is that ‘biomarkers’ identified using resting-state fMRI are difficult to interpret in terms of treatment (or disorder) mechanism, because the specific neural functions supported by at-rest activation and co-activation are poorly understood. Arguably, it is also difficult to distinguish them from non-neural differences in neurovascular coupling that affect resting state signal (e.g. psychotropic medication) [55]. This limits the interpretability of resting state co-activations, although does not necessarily reduce the potential prognostic value of these measures for brain stimulation response. Nevertheless, multivariate classifiers (and other data-driven approaches) tend to be agnostic about the mechanistic underpinnings of treatment response [18•]. Thus, data-driven approaches may be very useful at identifying what might predict response to brain stimulation, but may be less useful in understanding why that particular measure relates to treatment response.

### Theory-Driven Approaches

An alternative approach is ‘theory-driven’ biomarker development [56]. Earlier insights from pharmacological fMRI indicate that employing specific cognitive measures is key to understanding the neural mechanisms of drug effects [57]. Studies reporting an amelioration of negative emotional bias in depression after acute administration of antidepressant medication (e.g. [58–60]) led to major theoretical developments in the field. The cognitive neuropsychological account of depression treatment posits that depression arises from compromises in multiple interacting cognitive systems. Antidepressants target low-level emotion and reward-processing systems, reducing bias toward negative information processing frequently observed in depressed patients [40, 58, 61, 62]. This negative bias is enhanced by deficits in non-emotional ‘cold’ cognitive circuitry, including attention and decision-making; together, these interacting neurocognitive factors make a patient susceptible to the top-down negative expectations about the world characteristic of depression and targeted by cognitive behavioural therapy [40, 58, 61, 62].

A number of brain stimulation studies have employed cognitive tasks at baseline to assess their value as prognostic biomarkers. Pooling data from several trials, one study reported better pre-treatment letter fluency predicted response to left DLPFC tDCS, interpreting pre-treatment letter fluency as a proxy for preserved activity in the left DLPFC [63]. If this interpretation were correct, one might also expect greater DLPFC engagement during an executive function task to be associated with treatment response. Our later trial confirmed this prediction: Baseline left DLPFC activation during working memory was associated with subsequent clinical response to tDCS and not sham stimulation [9••]. In the context of the cognitive neuropsychological model of depression, this might suggest that tDCS, like CBT and potentially TMS [62], treats depression via alterations in ‘top-down’ cognitive control or emotion regulation, in contrast to antidepressant drugs, which are thought to directly alter negative emotional biases [39, 62]. This hypothesis is supported by preclinical evidence against an effect of tDCS on acute emotion perception [64]—note, however, that other work suggests that there may be an acute effect of tDCS on certain emotion-related processes such as threat vigilance [65].

Collecting a baseline index of the processing integrity of the stimulated region in a brain stimulation trial might have clinical utility (if replicated in larger trials). But more immediately, it can yield immediate mechanistic insight into the cognitive processes altered by the intervention. Such mechanistic insights from theory-driven biomarker approaches could simultaneously indicate ways to improve treatments for future trials. If, for example, greater DLPFC activation is required to obtain clinical response to DLPFC tDCS, increasing engagement of this cognitive system during or preceding tDCS delivery could increase a patient’s likelihood of response (e.g. by staircasing the difficulty of a cognitive task administered concurrently with tDCS to ensure that it adequately engages that individual’s DLPFC or by combining DLPFC tDCS and another intervention, such as rTMS) [66].

## A Pragmatic Framework for Biomarker Discovery

**Fig 1:**
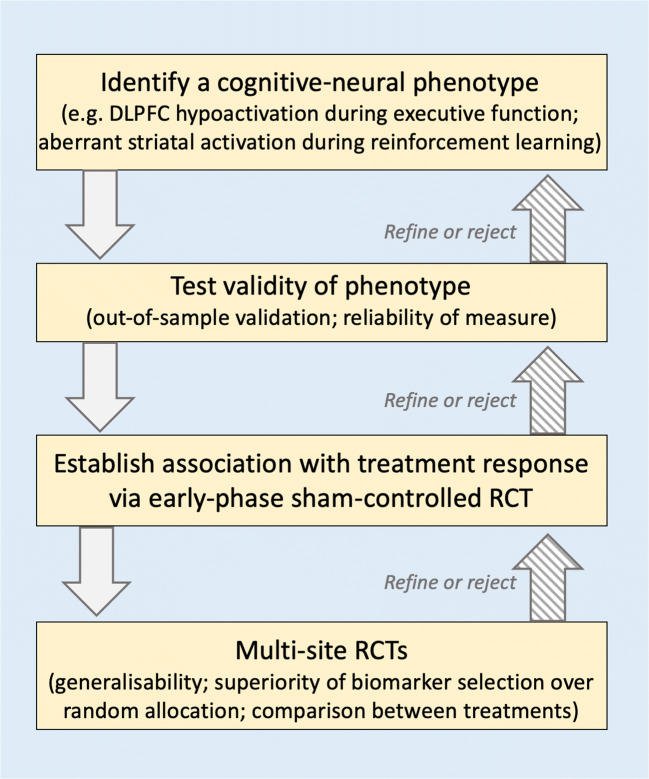
A pragmatic framework for identifying and testing brain stimulation biomarkers

How should we evaluate the clinical utility of such a wide array of putative anatomical and functional neural biomarkers? Numerous studies using a pre-post-trial design have reported neural or biological measures that correlate with treatment response. But currently, there is no established clinical biomarkers of treatment response for any type of brain stimulation. This is due to several barriers to establishing a neural measure as a biomarker, all of which must be addressed by any pragmatic framework (Fig. 1).

The first and perhaps the most difficult hurdle to overcome is identification of a reliable neural measure to modify with brain stimulation. I have argued here that depression itself does not have a reliable neural phenotype, so optimising brain stimulation for depression as a whole might be an impossible task. Instead, initial discovery research is needed to identify clusters of patients with a particular phenotype (as in the canonical correlation analysis approach by Drysdale and colleagues [50••]). However, these clusters must be stable. In the case of multivariate, data-driven methods used to identify a phenotype, the method must be validated using out-of-sample testing (see [53•] for recommendations for canonical correlation analysis). Even using simpler methods, such as univariate fMRI, stability of measurement is essential. Within-subject reliability of some neural activation measurement varies substantially according to imaging method, analysis approach, and region measured (see [67–69, 70•, 71, 72•]). Therefore, measurement reliability should be considered an essential facet of a putative neuromodulation biomarker [73] (note within-subject reliability is still only infrequently assessed in the context of randomised controlled trials suggesting putative brain stimulation biomarkers (RCTs) [9••]).

This phenotype-based approach would address another recent critique: that neuromodulation clinical trials fail because of issues with commonly used outcome measures, rather than due to a failure of the intervention itself [74••]. According to an elegant argument for improved primary outcome measures in brain stimulation trials, typical verbal report scales (e.g. Hamilton Depression Rating Scale (HAM-D [75]) or Montgomery-Asberg Depression Rating Scale (MADRS [76]) may fail to detect important clinical changes on relevant unmeasured clinical areas such as negative self-talk, optimism, and self-confidence, particularly when assessed only at one time point [74••]. Crucially, typical scales also do not easily dissociate separable components of depression (e.g. anhedonia; emotional dysregulation), despite their relatively distinct neural bases [77, 78]; these subcomponents might be more tightly coupled with treatment response than the full diagnostic criteria. Among other possible solutions, the authors propose measuring the effects of brain stimulation interventions on behavioural or symptom measures with a known neural circuitry. Instead of measuring the effects of an intervention on the entire major depressive disorder, trials could target specific neural circuits with a given intervention and measure change on associated behavioural or symptom outcomes [74••].

By establishing a stable and reliable neural phenotype or dimension, subsequent studies could then test two key factors: the phenotype’s association with treatment response, and its modifiability with particular brain stimulation interventions. Most of the studies reviewed here are examples of the first type of test. For the second, reliable data-driven approaches could identify dimensions that cut across diagnostic groupings, with experimental medicine studies developed to target this particular dimension. In one initial example of this translation from discovery science to experimental medicine, a psychiatric dimension related to disorders of compulsivity [79, 80], measured using a computationally derived measure of behaviour with a well-characterised neural basis [81, 82], was shown to be modifiable using cortico-cortico paired associative stimulation [83].

Finally, two types of randomised controlled trial (RCT) are required to test the specificity, utility, and validity of any putative biomarker. In the first type, an early-phase RCT is required to establish its specificity, at a minimum compared to its ability to predict response to sham stimulation, but ideally, compared to its ability to predict response to other interventions. In the second, multi-site trials are required to confirm the generalisability of the biomarker for prediction of clinical response. The utility of out-of-sample testing has been neatly demonstrated in the case of measuring sgACC activation to predict response to cognitive therapy for depression. In one trial, emotion-related sgACC deactivation was measured in two independent cohorts, before a cognitive therapy intervention (along with a third control cohort) [15]. Using this design, the researchers were able to predict response/remission in the second cohort based on activation thresholds obtained from the first, achieving over seventy per cent accuracy.

In summary, development and testing of brain stimulation biomarkers for depression requires: (1) identification of a cognitive-neural phenotype; (2) establishing its validity as putative biomarker, including out-of-sample replicability and within-subject reliability; (3) establishing the association between this phenotype and treatment response and/or its modifiability with particular brain stimulation interventions via an early-phase RCT; and (4) multi-site RCTs of one or more treatment types measuring the generalisability of the biomarker and confirming the superiority of biomarker-selected patients over randomly allocated groups.

## Conclusions

This framework provides an outline of how the neuromodulation field might develop and test putative neural biomarkers for treatment response in depression. However, neural biomarkers are not the only route to treatment prediction. Other treatments in psychiatry have used theory-driven approaches, such as performance on a cognitive task, or data-driven approaches on clinical and demographic measures to predict a patient’s likelihood of responding to antidepressant drugs [84] or cognitive behavioural therapy [85]. Both of these approaches to personalised psychiatry also have potential in neuromodulation, particularly if they are used as proxy measures for a neural phenotype which can then be directly targeted with brain stimulation. Outside of the brain, biological measures such as heart rate deceleration during initial rTMS delivery [86] may also hold promise as putative biomarkers. As novel forms of brain stimulation such as transcranial ultrasound stimulation begin translation to human patient studies, initial RCTs should incorporate potential biomarkers when establishing clinical effects, testing predictors of treatment response alongside group-level efficacy. Incorporation of potential biomarkers into RCTs could complement other innovation in trial design, such as updated measures of efficacy and outcome intended to better capture clinically meaningful change [74••]. By integrating the principles of dimensional psychiatry with improved trial designs that test reliability and generalisability, the field could move toward brain stimulation interventions designed to target specific neurocognitive phenotypes in depression.
